# Cold pressor‐induced sympathetic activation blunts the femoral but not carotid artery vascular responsiveness

**DOI:** 10.14814/phy2.70281

**Published:** 2025-03-20

**Authors:** Guilherme F. Speretta, Gaia Giuriato, Gianluigi Dorelli, Chiara Barbi, Anna Pedrinolla, Massimo Venturelli

**Affiliations:** ^1^ Department of Physiological Sciences, Biological Sciences Center Federal University of Santa Catarina Florianopólis Brazil; ^2^ Department of Neurosciences, Biomedicine and Movement Sciences University of Verona Verona Italy; ^3^ Surgical, Medical and Dental Department of Morphological Sciences Related to Transplant, Oncology and Regenerative Medicine University of Modena and Reggio Emilia Modena Italy; ^4^ Department of Cellular, Computational and Integrative Biology – CIBIO University of Trento Trento Italy; ^5^ Department of Internal Medicine University of Utah Salt Lake City Utah USA

**Keywords:** blood flow, blood pressure variability, cold pressor test, hemodynamics, vascular conductance

## Abstract

Vascular responsiveness due to passive leg movement (PLM) on the brain remains unknown. This study aimed to evaluate the effects of cold‐induced sympathetic activation (CPT) on femoral and ipsilateral and contralateral carotid arteries' vascular responsiveness evoked by PLM. Thirteen participants (seven males and six females; age: 27.0 ± 2.3 years) undertook a randomized session in which PLM was performed on the right leg at rest and during CPT. Right femoral (fBF) and right (ipsilateral) and left (contralateral) carotid (cBF) blood flows were measured by ultrasounds, and heart hemodynamics were assessed via photoplethysmography and impedance cardiograph. Systolic arterial pressure (SAP) time series were used to infer sympathetic modulation to the vessels. Femoral (fVC) and carotid (cVC) vascular conductance (BF/MAP) were calculated. CPT evoked changes in PLM on cBF, fBF, and fVC (interaction and time effect). cBF peak and cBF and cVC area under the curve were higher in the contralateral carotid in the two interventions. Low‐frequency power of SAP was higher in PLM‐CPT than in PLM; all *p* < 0.05. These results suggest that the CPT‐induced increases in sympathetic modulation attenuate the vascular responsiveness in the femoral, but not the carotid, arteries. Also, the contralateral carotid increased blood flow during PLM, regardless of the CPT.

## 1 INTRODUCTION

Maintaining adequate blood flow at rest and during physiological stressful events is mandatory to meet tissue metabolic demands (e.g., muscles and brain) (Andersen & Saltin, 1985; Caru et al., 1992; Joyner & Casey, 2015). This physiological equilibrium hinges on the intricate interplay between neural and local factors. The autonomic nervous system, alongside local vasoconstrictors and vasodilators, collaboratively determines the dynamic patterns of blood flow in different vascular beds; however, these patterns seem to be vessel‐specific (Hellsten et al., 2012; Peace et al., 2018; Pouwels et al., 2019).

The peripheral arteries' blood flow is mainly regulated by endothelium‐released factors, particularly nitric oxide (NO) (Joannides et al., 1995; Kooijman et al., 2008), while the sympathetic nervous system (SNS) plays a supporting role (Hellsten et al., 2012). On the contrary, the SNS seems to play a crucial role in the carotid arteries' blood flow regulation (Van Mil et al., 2018). Indeed, evidence supports that, in healthy individuals, the cold temperature‐induced carotid vasodilation (Peace et al., 2020) is likely primarily mediated by the SNS activation (Van Mil et al., 2018). The increased blood flow to the brain induced by the carotid vasodilation contributes to an acute regulation of vascular tone, ensuring proper blood supply during fluctuations in blood pressure and meeting the brain's energy needs in stressful situations (Fernandes et al., 2016). Notably, it seems that the SNS response in peripheral and central arteries depends on the receptor type, with the activation of α‐receptors on the smooth muscle and endothelium leading to vasoconstriction, while the activation of β‐receptors in the endothelium induces vasodilation (Hellsten et al., 2012; Peace et al., 2018; Pouwels et al., 2019).

Among the techniques to induce detectable vascular responsiveness in humans, the passive leg movement (PLM) (Groot et al., 2015; Mortensen et al., 2012) has been increasingly used due to its relatively easy implementation and correlation with traditional tests, such as flow‐mediated dilation (Rossman et al., 2016). Indeed, it has been shown that NO release is involved in the transitory (~5–20 s to reach the peak and ~35–45 s to return to baseline) increase in femoral artery blood flow evoked by the PLM, likely stimulated by the repeated muscle length changes during the passive muscle movement (Clifford et al., 2006). Importantly, the continuous PLM evokes pronounced central hemodynamic responses, while single PLM produces a minimal effect (Broxterman et al., 2017; Venturelli, Layec, et al., 2017).

A recent study has shown that vascular responsiveness is attenuated by exercise‐induced increases in muscle sympathetic nerve activity, emphasizing the role of autonomic modulation of the skeletal muscle vascular conductance (Venturelli et al., 2022). The cold pressor test (CPT) also evokes a sympathetic activation (Victor et al., 1987) without significant metabolic and temperature perturbations, promoting alterations in vascular responsiveness. Specifically, CPT induces vasodilation in the carotid artery in healthy participants (Van Mil et al., 2018; Peace et al., 2020), while the results for the brain vasculature are contrasting. For instance, Studies show that CPT evoked increases in cerebral blood flow (Flück et al., 2017; Tymko et al., 2017), while others have not found changes (Washio et al., 2020).

On the other hand, no studies have described the carotid vascular responsiveness to PLM and the concomitant responses in peripheral and central hemodynamics. In this sense, it has been demonstrated that the mechanical stimulation induced by the PLM evokes a remote effect (i.e., increased blood flow) in the contralateral leg (McDaniel et al., 2010; Wray et al., 2005), which may be related to a reflex‐induced sympathetic activity reduction and a consequent decrease in systemic vascular resistance in the contralateral leg, as observed during voluntary exercise in resting limbs (Callister et al., 1994). Voluntary contraction also increases blood flow in the contralateral carotid artery that supplies the cortical representation of the arm to increase oxygen delivery (Fernandes et al., 2016). This response is, at least in part, due to neurovascular coupling that reduces sympathetic vasoconstriction. At the same time, it limits blood flow changes in the ipsilateral carotid artery (Fernandes et al., 2016).

Therefore, evaluating the ipsilateral and contralateral carotid arteries vascular responsiveness to PLM is relevant to understanding whether the blood flow to the brain is also remotely impacted by the PLM procedure. This study aimed to evaluate the vascular responsiveness to PLM on the ipsilateral and contralateral carotids and femoral artery. The combination of PLM with the cold‐induced sympathetic activation on the vascular responsiveness of femoral and carotid arteries was also investigated. We hypothesized that PLM would remotely increase the contralateral, but not the ipsilateral, carotid artery blood flow. In addition, the cold‐induced sympathetic activation would attenuate the femoral artery and the carotid arteries vascular responsiveness.

## 2 MATERIALS AND METHODS

### 2.1 Participants characteristics

Thirteen healthy young participants (seven males and six females; age: 27 ± 2 years; body mass: 72 ± 21 Kg; and height 1.69 ± 0.12 m; mean ± SD) were recruited and took part in the study. Exclusion criteria were as follows: have smoked in the last 6 months; have visible or known cardiovascular, metabolic, or infectious diseases. The autonomic modulation data of one participant were excluded due to problems with data acquisition. Therefore, data from 12 participants are reported for this variable. The ethical committee approved all procedures of the University of Verona (CARP; acceptance number IRB #27111) and were performed following the Declaration of Helsinki standards. The participants gave written, informed consent before their participation after a full explanation of the purpose and experimental procedures of the study.

The sample size was determined using an α of 0.05, a power (1‐β) of 0.80, and an effect size of 0.87, based on the PLM‐induced vascular conductance increases during PLM alongside SNS activation, as reported in a previous study (Venturelli et al., 2022). The calculation indicated the need for 11 participants. Pondering a 10% loss rate, the final sample was adjusted to 13.

### 2.2 Experimental design

**FIGURE 1:**
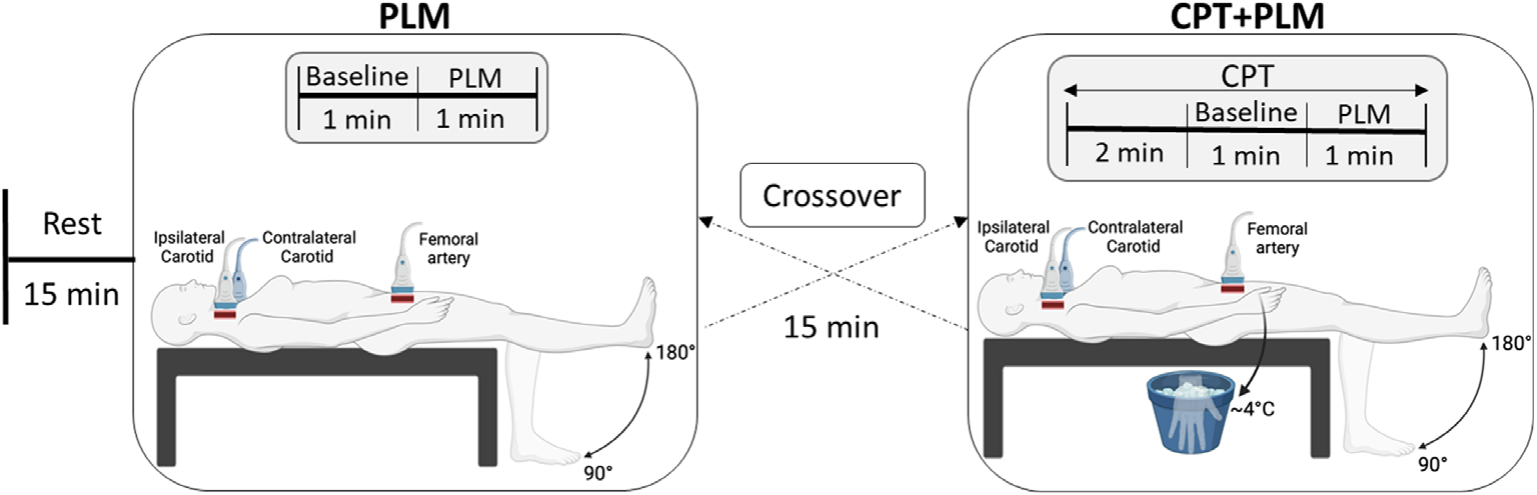
Experimental design. CPT, cold pressor test; PLM, passive leg movement.

The participants attended the laboratory once for evaluations in this randomized crossover study (Figure 1). We asked them to abstain from intense exercise and caffeine and antioxidants consumption for 24 h before the experiments. The participants arrived in the morning (9–11 am) in a fasted state (at least 6 h). After 15 min of resting baseline measurements, they underwent two experimental conditions separated by 15 min of rest. Specifically, condition A (PLM) consisted of 60 s of baseline followed by PLM, while condition B (CPT + PLM) consisted of a 3‐min cold pressor test (CPT) followed by 60 s of combined CPT and PLM. The intervention order was randomized and counterbalanced among participants. We used three ultrasound Doppler systems to evaluate the diameter and blood velocity of the right femoral artery and the right (ipsilateral) and left (contralateral) carotid arteries before and during the tests. Blood pressure, stroke volume (SV), cardiac output (CO), heart rate (HR), and pulse intervals were also recorded in the same periods. All the tests were performed in a supine position. We described the method's details below.

### 2.3 Cold pressor test

The CPT was performed with the participants positioned close to the right side, ensuring hand movement without considerably moving the neck. During CPT, the right hand was submerged up to the wrist in cold water (~4°C) for 4 min. The temperature was monitored with a real‐time digital thermometer (range: −50°C – +300°C). Participants were instructed to breathe normally and not speak during the test (Peace et al., 2020). All the participants completed the task without relating any side effects.

### 2.4 Passive leg movement

Each protocol consisted of 60 s of passive rhythmic knee flexo‐extension. PLM was performed by a researcher who moved the participant's right lower leg through a 90° range of motion from full extension (180°) to a 90° knee joint angle at 1 Hz (Venturelli et al., 2022). We used a metronome to perform extension and flexion at the established rate. Before starting PLM and throughout the test, the researcher instructed participants to avoid tensing their leg muscles to accelerate or decelerate the movement.

### 2.5 Carotid and femoral blood flow and vascular conductance

Arterial blood velocity and vessel diameter measurements were continuously and simultaneously achieved in the ipsilateral and contralateral carotids and right femoral arteries. Specifically, both right and left common carotids, ~2 cm proximal from the carotid bulb, were scanned with 2 Logiq‐e ultrasound Doppler systems (General Electric Medical Systems, Milwaukee, WI) (Peace et al., 2020). The femoral artery of the passively moved limb was scanned distal to the inguinal ligament and proximal to the deep and superficial femoral bifurcation with a Logiq‐7 ultrasound Doppler system (General Electric Medical Systems, Milwaukee, WI) (Venturelli, Layec, et al., 2017). The ultrasound Doppler systems were equipped with a 12‐ to 14‐MHz linear array transducer. For all arteries, the diameter was determined at a 90° angle along the central axis of the scanned area. Mean blood velocities (V_mean_) were measured using the same probes with a 5‐MHz frequency. Measurements of V_mean_ were obtained with the probes positioned to maintain an insonation angle of 60°, and the sample volumes were centered and maximized according to the vessels' size. Utilizing the arterial diameter and the V_mean_, the blood flow (BF) was calculated second by second as:

$$\mathrm{BF}=V_{\text{mean}}\times \pi \times {\left(\frac{\;\text{Vessel Diameter}}{2}\;\right)}^{2}\times 60$$

where BF is in milliliters per minute (Venturelli, Layec, et al., 2017). Experienced and skilled sonographers performed scanning and analyses.

### 2.6 Cardiovascular parameters

Beat‐by‐beat arterial systolic (SAP) and diastolic (DAP) arterial pressure were determined by finger plethysmography (Finapres model 2300; Ohmeda, Englewood, CO, USA). The photoplethysmography cuff of the finger pressure device was placed on the left hand's third finger. The Finapres signal was calibrated using the rest blood pressure obtained by a sphygmomanometer. All the signals were recorded with the Power Lab System (PowerLab 16/30; ML880, ADInstruments, Bellavista, NSW, Australia). A noninvasive thoracic impedance cardiograph (Physio Flow®, Manatec, Strasbourg, France) was used to measure HR and estimate SV.

### 2.7 Data collection and analysis

V_mean_ of the femoral and carotid arteries was analyzed with 1 Hz resolution on the Doppler ultrasound system at baseline (30 s) and during PLM (60 s). Before analysis, all hemodynamic data were smoothed using a 3‐s rolling average (Venturelli, Cè et al., 2017). As the response to PLM is transient and varies between individuals, a peak response was determined individually for all variables. Maximal relative changes (∆peak) were calculated as the peak (negative or positive) minus the baseline, and area under the curve (AUC) was determined after normalization for baseline as the summed response for 60 s (Broxterman et al., 2017) for each participant in all measured variables (Venturelli et al., 2019). Mean arterial blood pressure (MAP) was calculated as (1/3 SAP +2/3 DAP) and expressed as mmHg. Femoral and carotids vascular conductance (fVC and cVC, respectively) were calculated as BF/MAP. CO was calculated as SV × HR (Richard et al., 2001).

Frequency‐domain analysis of the SAP was performed before and during PLM by custom computer software (CardioSeries v2.4, http://www.danielpenteado.com). Briefly, time series were transformed into evenly spaced series by cubic spline interpolation (4 Hz) and were distributed into half‐overlapping sets of 300 points (Welch periodogram). A Hanning window was used to attenuate side effects, and the interpolated time series had the spectra calculated by the Fast Fourier Transform algorithm. Then, the spectra were integrated into different frequencies (Formolo et al., 2022; TASK FORCE, 1996). The low‐frequency (LF; 0.04–0.15 Hz) power (mmHg^2^) was used to infer the sympathetic modulation to the vessels (Formolo et al., 2022; Speretta et al., 2016).

### 2.8 Statistical analysis

The student's paired *t*‐test was used to determine the differences in cardiovascular and sympathetic reactivity (∆ Peak and AUC) between PLM and CPT + PLM. Analysis of variance (ANOVA) with repeated measures followed by Bonferroni's post‐hoc test was applied for cardiovascular reactivity to PLM as a function of time. All data were analyzed using a statistical software package, Graph Pad Prism v.6 (GraphPad Software, San Diego, California, USA). Data are presented as mean ± standard deviation (SD) and considered significant when *p* < 0.05.

## 3 RESULTS

### 3.1 Carotid arteries reactivity to PLM and PLM + CPT

**FIGURE 2:**
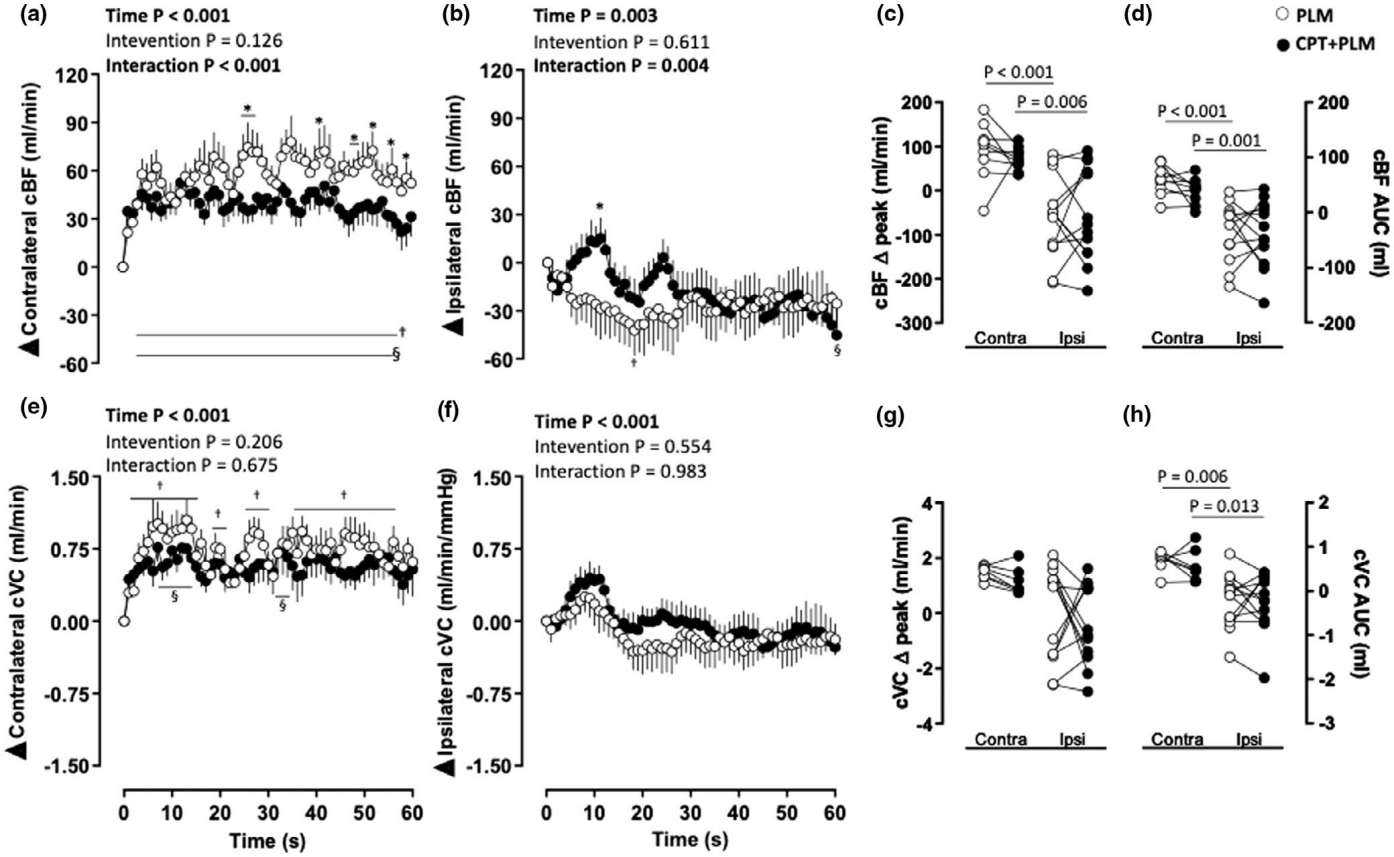
(a) Contralateral (Contra) and (b) Ipsilateral (Ipsi) carotid blood flow (cBF) as a function of time, (c) delta peak, and (d) delta area under the curve (AUC); (e) contralateral and (f) ipsilateral carotid vascular conductance (cVC) as a function of time, (g) delta peak, and (h) delta AUC in response to passive leg movement (PLM) at rest and during the cold pressor test (CPT). Analysis of variance (ANOVA) with repeated measures followed by Bonferroni's post hoc test for panels (a) and (d); Student's paired *t*‐test for panels (b), (c), (e), and (f); *p* < 0.05; Significant results for ANOVA are highlighted in bold; † vs. baseline at PLM; § vs. baseline at CPT + PLM; * vs. PLM; *n* = 13 for ipsilateral cBF and cVC data; *n* = 9 for contralateral cBF data; *n* = 7 for contralateral cVC data.

The PLM evoked changes in the contralateral cBF over time, which were impacted by the CPT (main time and intervention × time interaction). Contralateral cBF increased from 3 s to 56 s and from 3 s to 54 s in PLM and CPT + PLM, respectively (*p* < 0.05; Figure 2a). Higher contralateral cBF was observed at 25–27, 35–37,42,46–47, 52, 56, and 59 s in the PLM compared to CPT + PLM (*p* < 0.05; Figure 2a).

The PLM also evoked changes in the ipsilateral carotid BF (cBF) over time, which were impacted by the CPT (main time and intervention effects and an intervention × time interaction). Decreases in ipsilateral cBF at 19 and 60 s were observed in PLM and CPT + PLM, respectively (*p* < 0.05; Figure 2b). Higher ipsilateral cBF was observed at 12 s in the CPT + PLM compared to PLM (*p* < 0.05; Figure 2b).

The ipsilateral cBF delta peak (*p* < 0.001 and *p* = 0.006; Table 1 and Figure 2c) and AUC (*p* < 0.001 and *p* = 0.001; Table 1 and Figure 2d) were lower compared to the contralateral in PLM and CPT + PLM.

**TABLE 1:** Peripheral and central hemodynamic and autonomic modulation during passive leg movement (PLM) and cold pressor test (CPT) + PLM.

Variable	PLM				CPT + PLM			
	Baseline	Peak	∆ peak	AUC	Baseline	Peak	∆ peak	AUC
Contralateral cBF (mL/min)	419 ± 132	511 ± 176	90 ± 65	58 ± 27	367 ± 130	440 ± 132	72 ± 26	39 ± 23
Contralateral carotid diameter (cm)	0.57 ± 0.08	0.58 ± 0.08	0.01 ± 0.02	–	0.56 ± 0.07	0.55 ± 0.08	−0.01 ± 0.04	–
Contralateral cVC (mL/min/mmHg)	4.6 ± 0.8	6.1 ± 1.0	1.4 ± 0.2	0.6 ± 0.2	3.9 ± 0.7	5.0 ± 1.0	1.1 ± 0.4	0.5 ± 0.3
Ipsilateral cBF (mL/min)	542 ± 140	479 ± 133	−63 ± 94	−34 ± 52	566 ± 176	511 ± 117	−54 ± 106	−38 ± 52
Ipsilateral carotid diameter (cm)	0.66 ± 0.07	0.67 ± 0.07	0.02 ± 0.03	–	0.69 ± 0.07	0.68 ± 0.8	−0.01 ± 0.03	–
Ipsilateral cVC (mL/min/mmHg)	6.1 ± 1.4	6.0 ± 1.9	−0.06 ± 1.73	−0.2 ± 0.6	5.7 ± 2.0	5.1 ± 1.7	−0.50 ± 1.43	−0.2 ± 0.6
fBF (mL/min)	341 ± 143	901 ± 243	560 ± 225	205 ± 78	461 ± 209	957 ± 563	495 ± 443	67 ± 151*
fVC (mL/min/mmHg)	3.9 ± 1.6	12.7 ± 3.8	8.8 ± 3.5	2.4 ± 0.8	4.7 ± 2.3	11.5 ± 6.9	6.6 ± 5.4*	0.8 ± 1.8*
MAP (mmHg)	88 ± 6	83 ± 9	−4.9 ± 6.4	−1.5 ± 2.4	101 ± 15	96 ± 15	−5.1 ± 9.3	−2.3 ± 4.0
Heart rate (bpm)	63 ± 11	71 ± 13	8.6 ± 6.0	3.4 ± 3.3	66 ± 12	74 ± 17	2.2 ± 13.9	−3.1 ± 7.6*
Cardiac output (L/min)	4.7 ± 1.1	5.1 ± 1.8	0.4 ± 1.9	0.20 ± 0.35	4.1 ± 1.3	4.4 ± 1.4	0.2 ± 1.4	−0.02 ± 0.42
Stroke volume (mL)	88 ± 24	93 ± 29	5.1 ± 17.8	−1.1 ± 6.0	80 ± 24	86 ± 32	5.7 ± 25.4	1.6 ± 6.6
LF SAP (mmHg^2^)	2.8 ± 1.91	2.1 ± 1.9	−0.6 ± 1.3	–	2.0 ± 1.9	5.2 ± 2.8	3.1 ± 3.3*	–

Abbreviations: AUC, area under the curve; cBF, carotid blood flow; cVC, carotid vascular conductance; fBF, femoral blood flow; fVC, femoral vascular conductance; MAP, mean arterial pressure.

* *p* < 0.05 versus PLM. Data are presented as mean ± standard deviation; *n* = 13, except for LF SAP (*n* = 12), contralateral cBF (*n* = 9), and contralateral cVC (*n* = 7).

The PLM also induced changes in the contralateral and ipsilateral cVC over time, but the response was not affected by the CPT (main time effect; Figure 2e,f). Increases in contralateral cVC were observed in PLM at 5–17, 20–21, 27–30, and 33–57 s and CPT + PLM at 7–13 and 33–34 s (*p* < 0.05; Figure 2e).

The ipsilateral cVC AUC (*p* = 0.006 and *p* = 0.013; Table 1 and Figure 2c) was lower compared to contralateral in PLM and CPT + PLM.

### 3.2 Femoral artery reactivity to PLM and PLM + CPT

**FIGURE 3:**
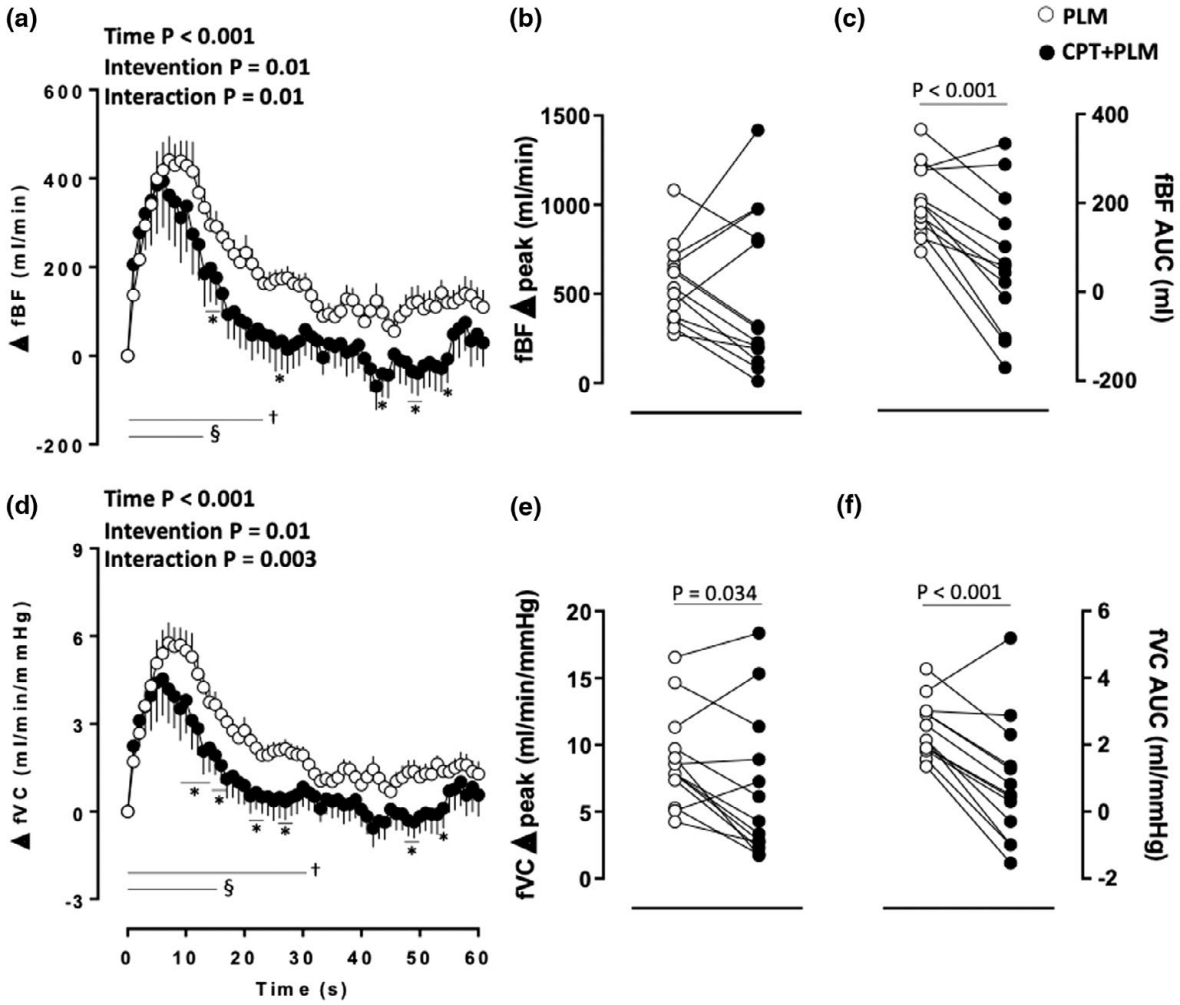
Femoral blood flow (fBF) (a) as a function of time, (b) delta peak, and (c) delta area under the curve (AUC); and femoral vascular conductance (fVC), (d) as a function of time, (e) delta peak, and (f) delta AUC in response to passive leg movement (PLM) at rest and during the cold pressor test (CPT). Analysis of variance (ANOVA) with repeated measures followed by Bonferroni's post hoc test for panels (a) and (d); Student's paired *t*‐test for panels (b), (c), (e), and (f); *p* < 0.05; Significant results for ANOVA are highlighted in bold; † vs. baseline at PLM; § vs. baseline at CPT + PLM; * vs. PLM; *n* = 13.

The PLM evoked changes in the femoral BF (fBF) over time, which were affected by CPT (main time and intervention effects and an intervention × time interaction). Increases from baseline in fBF from 1 to 31 s and 1 to 15 s were observed in PLM and CPT + PLM, respectively (*p* < 0.05). Higher fBF was observed at 21, 22, 28, 43, 49, 50, and 54 s in the PLM compared to CPT + PLM (*p* < 0.05; Figure 3a). The fBF delta AUC was higher in the PLM compared to CPT + PLM (*p* < 0.001; Table 1 and Figure 3c). No differences between interventions were observed in the fBF delta peak (*p* = 0.50; Table 1 and Figure 3b).

The PLM also induced changes in the fVC over time, which were affected by CPT (main time and intervention effects and an intervention × time interaction). Increases from baseline in fVC from 1 to 31 s and 1 to 16 s were observed in PLM and CPT + PLM, respectively (*p* < 0.05; Figure 3d). Higher fVC was observed from 9 to 14, 16 to 18, 21 to 22, 26 to 28, 43, 49 to 50, and 54 s in the PLM compared to CPT + PLM (*p* < 0.05; Figure 3d). The fVC delta peak (*p* = 0.034; Table 1 and Figure 3e) and AUC (*p* < 0.001; Table 1 and Figure 3f) were higher in the PLM when compared to CPT + PLM.

### 3.3 Central hemodynamics and sympathetic modulation reactivity to PLM and PLM + CPT

**FIGURE 4:**
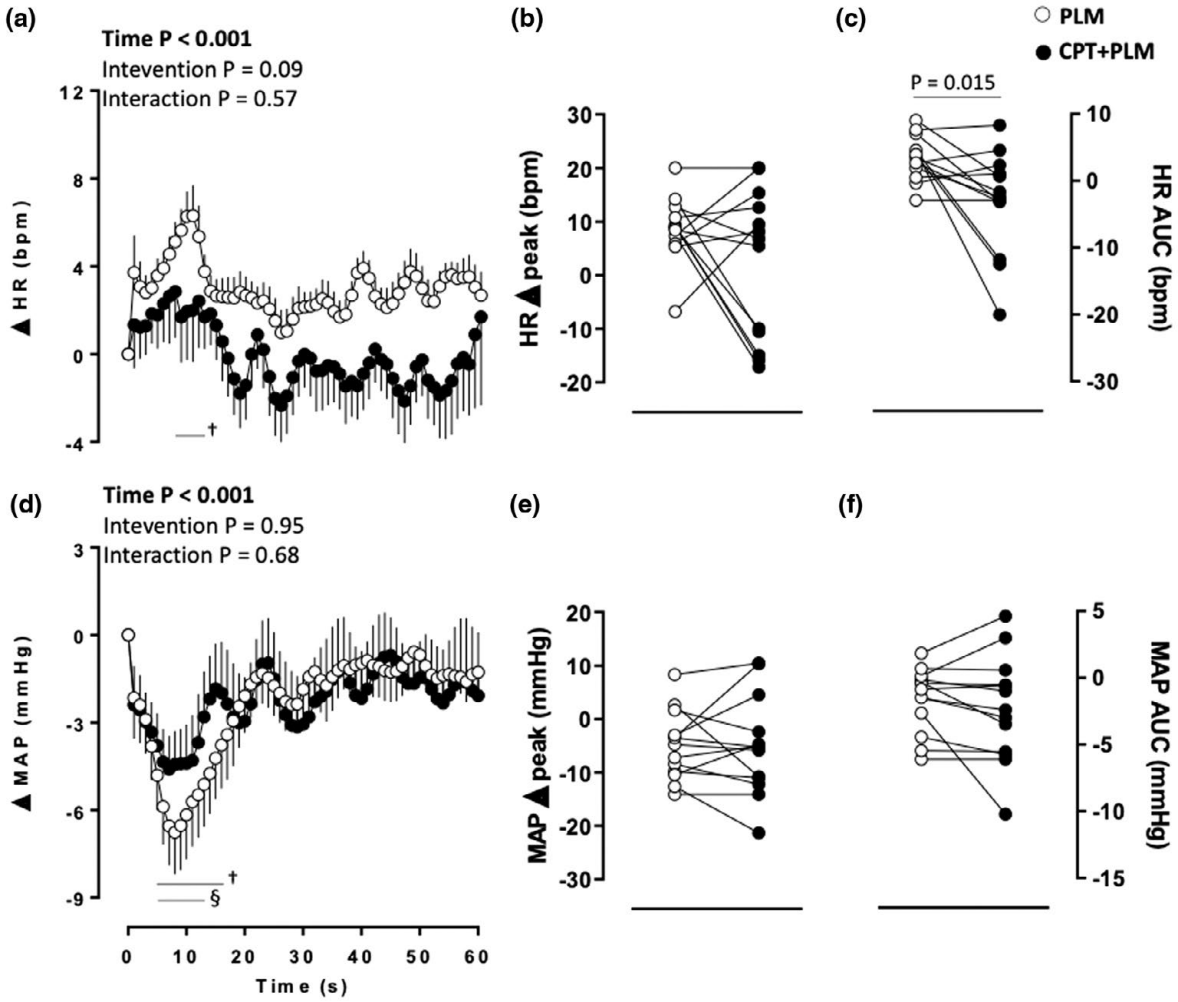
Heart rate (HR) (a) as a function of time, (b) delta peak, and (c) delta area under the curve (AUC); and mean arterial pressure (MAP), (d) as a function of time, (e) delta peak, and (f) delta AUC in response to passive leg movement (PLM) at rest and during the cold pressor test (CPT). Analysis of variance (ANOVA) with repeated measures followed by Bonferroni's post hoc test for panels (a) and (d); Student's paired *t*‐test for panels (b), (c), (e), and (f); *p* < 0.05; Significant results for ANOVA are highlighted in bold; † vs. baseline at PLM; § vs. baseline at CPT + PLM; *n* = 13.

The PLM induced changes in the HR over time, but it was not affected by the CPT (main time effect). Increases in HR from 9 to 13 s were observed in PLM (*p* < 0.05; Figure 4a). The HR AUC was higher in the PLM than CPT + PLM (*p* = 0.015; Table 1 and Figure 4c). No differences between interventions were observed in the HR delta peak (*p* = 0.15; Table 1 and Figure 4b).

The PLM also evoked changes in the MAP over time, which was not impacted by the CPT (main time effect). Decreases in MAP from 5 to 17 s and from 5 to 13 s were observed in PLM and CPT + PLM, respectively (*p* < 0.05; Figure 4d). The MAP delta peak (*p* = 0.91; Table 1 and Figure 4e) and AUC (*p* = 0.32; Table 1 and Figure 4f) showed no differences between interventions.

**FIGURE 5:**
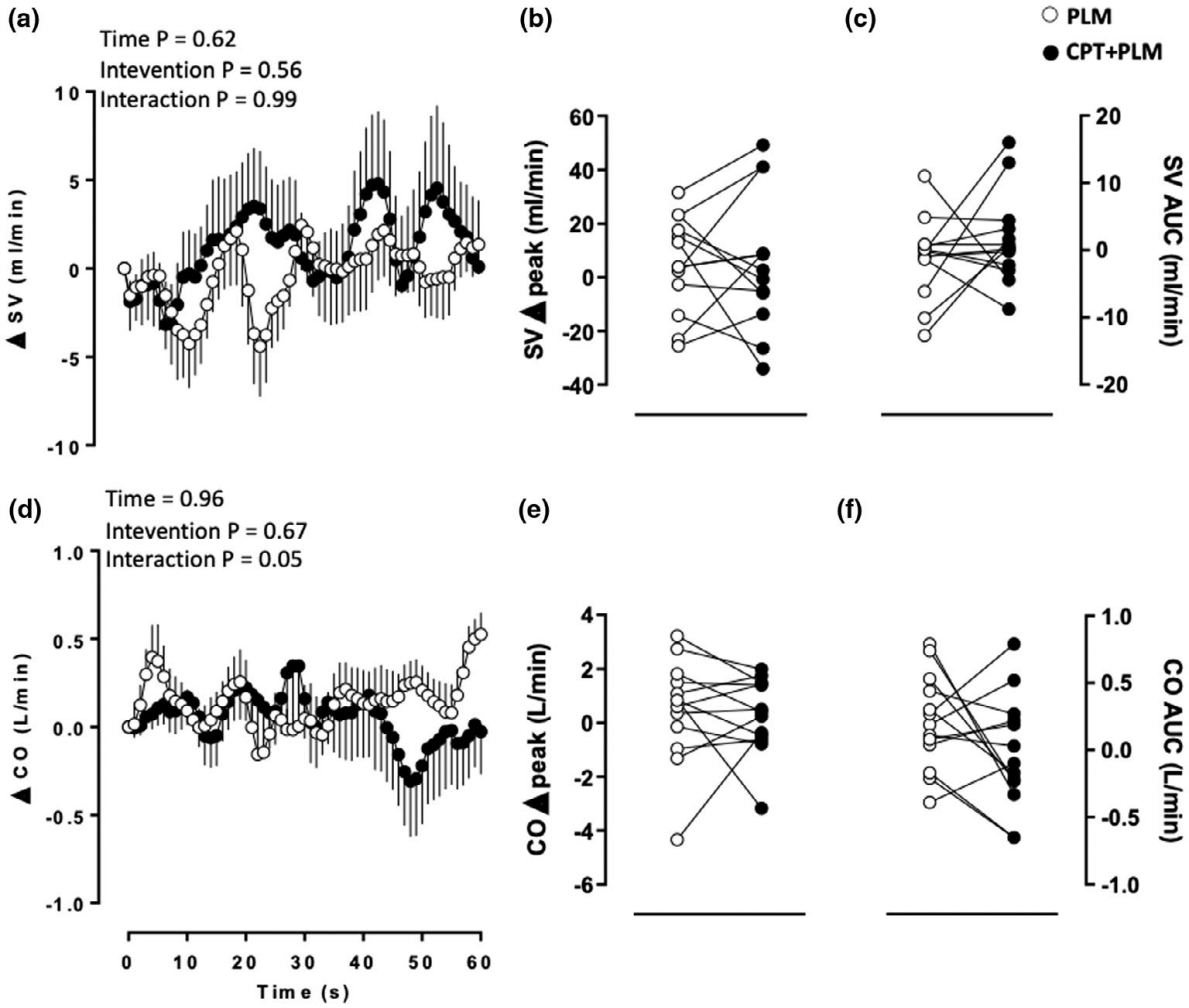
Stroke volume (SV) (a) as a function of time, (b) delta peak, and (c) delta area under the curve (AUC); and cardiac output (CO), (d) as a function of time, (e) delta peak, and (f) delta AUC in response to passive leg movement (PLM) at rest and during the cold pressor test (CPT). Analysis of variance (ANOVA) with repeated measures followed by Bonferroni's post hoc test for panels (a) and (d); Student's paired *t*‐test for panels (b), (c), (e), and (f); *p* < 0.05; *n* = 13.

The SV and CO reactivity analysis showed no effects. The SV and CO delta peaks (*p* = 0.93 and *p* = 0.72, respectively) and AUC (*p* = 0.33 and *p* = 0.12, respectively) showed no differences between interventions (Table 1 and Figure 5).

The LF power reactivity was higher in CPT‐PLM than in PLM (*p* < 0.01; Table 1).

### 3.4 Absolute values of carotid and femoral arteries, central hemodynamics, and sympathetic modulation

The absolute values of carotid and femoral arteries, central hemodynamics, and LF power are described in Table 1.

## 4 DISCUSSION

The present study investigated the vascular responsiveness due to PLM at peripheral (femoral artery) and brain (both carotid arteries) levels. Potential interactions between sympathetic activation (cold‐pressor exposure) and the PLM‐induced changes in vascular responsiveness were also investigated. PLM evoked increases only in the contralateral carotid artery vascular responsiveness, and the CPT did not change the response. Additionally, PLM‐induced vascular responsiveness was attenuated by CPT at the femoral artery level. Finally, we found higher sympathetic modulation to the vessels during CPT + PLM than PLM.

### 4.1 Carotid arteries vascular responsiveness to PLM at rest and during CPT

To our knowledge, this is the first study evaluating the carotid vascular responsiveness due to the PLM. We did not observe important alterations in ipsilateral cBF and cVC during PLM and CPT + PLM, while the contralateral cBF increased during both interventions, which suggests an ability to maintain blood flow and meet the brain metabolic demands in a challenging scenario that includes a fast blood flow redistribution (i.e., increases in fBF) and the consequent blood pressure decrease. Although it is currently unknown to what extent cerebral metabolic control contributes to the regulation of carotid artery vasomotor function (Peace et al., 2018), dilatation of the internal carotid artery during physiological challenges (i.e., hypercapnia) (Carter et al., 2016; Hoiland et al., 2016) has been observed. Increases in shear stress seem to trigger this response (Carter et al., 2016). Also, the increase in the blood flow only in the contralateral carotid is in line with the response observed during handgrip exercise for both carotid (Fernandes et al., 2016) and middle cerebral (Pott et al., 1997) arteries. The mechanism involved in the lateral‐dependent response may be related to the neurovascular coupling (Fernandes et al., 2016). Indeed, during voluntary contraction, the SNS mediates the reduction in the ipsilateral and increase in the contralateral carotid blood flow supplying the active limb's cortical area (Fernandes et al., 2016).

During CPT + PLM, we observed a higher LF power of the SAP reactivity compared to PLM, suggesting a higher SNS modulation to the vessels. Unlike peripheral vessels, the increased SNS modulation to the carotid appears to promote higher carotid hyperemia (Rubenfire et al., 2000). Although the mechanisms of this response are not completely understood, it is suggested that CPT‐induced changes in carotid artery hyperemia are related to an SNS effect on specific adrenoreceptors expressed in the vascular smooth muscle and endothelial cells (Peace et al., 2018; Pouwels et al., 2019). Specifically, during CPT, the activation of the endothelium β1‐, β2‐, β3‐, and α2‐receptors and smooth muscle cells β1‐ and β2‐receptors leads to vasodilation, which opposes the vasoconstriction stimulus induced by the α1‐ and α2‐receptors activation on the smooth muscle cells (Peace et al., 2018). Thus, the balance of those processes is responsible for the vasoreactivity of the common carotid artery (Pouwels et al., 2019; Rubenfire et al., 2000). Although we did not observe a carotid dilation during CPT‐PLM, it also did not constrict, suggesting a role of the SNS in the maintenance of cBF and cVC. Previous studies have demonstrated that the peak reactivity of carotid diameter to CPT usually occurs around 60–120 s into the test, with the variables tending to return to baseline values thereafter (Peace et al., 2020). Our decision to commence PLM in the fourth minute of CPT may explain the absence of carotid dilation.

It is also important to note that cerebral blood flow regulation, an intrinsic mechanism that maintains adequate cerebral perfusion in dynamic or spontaneous blood pressure changes (Claassen et al., 2021; Saleem et al., 2018), may partially explain the cBF reactivity during PLM and CPT + PLM. Previous data demonstrated that CPT did not change cerebral autoregulation (Washio et al., 2020). Also, given the significant variation in blood pressure during PLM, it is reasonable to propose that the baroreflex is involved in the carotid responsiveness. However, the activation of the afferent pathways from the mechanoreceptors in the joints and muscles appears to be more prominent (McDaniel et al., 2010).

### 4.2 Effect of CPT on femoral artery vascular responsiveness due to PLM

It is well established that, in peripheral arteries (e.g., femoral and brachial), the vascular responsiveness induced by the PLM is mainly mediated by local factors, especially NO (Joannides et al., 1995; Kooijman et al., 2008). Specifically, ~70% of the PLM‐induced vascular responsiveness is NO‐mediated (Groot et al., 2015; Trinity et al., 2012). Nevertheless, preclinical and clinical studies suggest that SNS activity influences fVC, especially during physiological stressful events, such as exercise (Peterson et al., 1988; Venturelli et al., 2022) and CPT (Miller et al., 2019). For instance, arm‐cranking exercise blunted femoral artery hyperemia induced by PLM in young, healthy people, which was associated with muscle SNS activity (Venturelli et al., 2022) and the acute lumbar sympathectomy increased the vascular conductance induced by exercise in rats (Peterson et al., 1988). In addition, a previous clinical study showed that CPT also increases the muscle SNS activity (Victor et al., 1987). These results align with our data since we demonstrated a reduction in PLM‐induced vascular responsiveness during the CPT, which was associated to an increase in the SNS modulation to the vessels. Specifically, we observed lower fBF and fVC during CPT + PLM compared to PLM. Notably, in our study, CPT was performed in the participant's hand while the vascular conductance was evaluated in the femoral artery, demonstrating a remote effect induced by the cold water. Thus, as observed during physical exercise (Venturelli et al., 2022; Wray et al., 2009), the higher SNS modulation evoked by the CPT may attenuate the NO‐induced vasodilation in response to the repeated muscle length‐dependent changes in vessel tortuosity during the PLM (Mortensen et al., 2012; Trinity et al., 2012).

We performed the protocol with the participants in the supine position to facilitate the carotid artery ultrasonography, acknowledging that this may have influenced the responses. Indeed, a greater (more than twofold) vascular responsiveness to PLM in the upright compared to the supine position has been observed (Trinity et al., 2011). Therefore, future studies should evaluate the effects of CPT on PLM in the upright position.

### 4.3 Effect of CPT and PLM on central hemodynamic

Previous studies have demonstrated marked PLM‐induced central hemodynamic responses (Groot et al., 2015; Trinity et al., 2012; Venturelli, Layec, et al., 2017). Accordingly, we found robust decreases in MAP at the beginning of PLM and CPT + PLM. On the other hand, we did not find CO and SV alterations in both interventions, which contrasts with previous studies that showed increases in CO and SV (Groot et al., 2015; Trinity et al., 2012; Venturelli, Layec, et al., 2017). Those previous studies were performed only in males, while in our study, almost half of the participants were females. Thus, differences in the amount of muscle mass and blood pressure regulation (Hart et al., 2009) between males and females may help explain these differences. Indeed, skeletal muscle afferent feedback plays a key role in the hemodynamic responses induced by PLM, and disparities in joint and muscle mechanoreceptors may change the magnitude of the response (Trinity et al., 2010). Regarding HR, we only observed a transient increase over time during PLM and higher AUC HR in the PLM compared to CPT + PLM. The difference in the HR AUC may be related to the HR at PLM and CPT + PLM baseline since there was a trend of higher values before CPT + PLM compared to PLM (*p* = 0.070). Also, it has been demonstrated that CPT evokes an increase in central hemodynamics in the initial period of the test (Victor et al., 1987). Although there is individual variability in the HR pattern during the CPT (Mourot et al., 2009), the peak hemodynamic response appears to be between the first and the second minute. Subsequently, MAP and HR tend to return to the baseline values (Peace et al., 2020), most likely due to a decrease in the SNS modulation and parasympathetic modulation reactivation (Mourot et al., 2009). Since the participants underwent PLM in the fourth minute of CPT, it is possible that the influence of CPT on autonomic modulation during CPT + PLM does not allow for the same pattern of response observed only in PLM. Indeed, the mechanoreflex activation due to PLM with and without the CPT can be seen in the heart chronotropic response (Table 1 and Figure 4a–c). Considering the concomitant changes in the contralateral cBF and cVC, it is reasonable to assume some remote mechanoreflex effects, likely driven by the parasympathetic modulation activation. Indeed, further studies are mandatory for addressing this matter, including the more specific measurements of sympathetic and parasympathetic activation during PLM and CPT.

### 4.4 Limitations

We did not evaluate sympathetic activity directly. Also, we did not consider the females' menstrual cycle. Nevertheless, the menstrual cycle seems to have a minor effect on endothelial function, which may be partly related to different methodological approaches (Williams et al., 2020). Furthermore, testing females only in the initial week of the cycle may reduce the ecological validity of the studies. Our sample size was insufficient to allow a comparison between males and females. We recommend that future studies consider conducting this analysis. Finally, we were not able to evaluate both carotid arteries' responsiveness in all the participants due to technical reasons. However, taking into account the methods used in the study (i.e., doppler analysis), we have reasonable data for the statistical analysis we performed.

## 5 CONCLUSION

Our results suggest that CPT reduces the femoral artery, but not the carotid artery, hyperemia evoked by the PLM. Furthermore, contralateral carotid increased blood flow during PLM regardless of the CPT. The maintenance of cBF and cVC during CPT + PLM seems to be associated with the CPT‐induced increases in sympathetic modulation to the vessels. These results may have clinical implications. Future studies should evaluate the effects of PLM on carotid blood flow at rest and during sympathetic activation in different populations, including older adults and people with chronic diseases.

## AUTHOR CONTRIBUTIONS

G.F.S. and M.V. conceived and designed the research, edited the manuscript, and interpreted the results of the experiments. G.F.S. analyzed data, prepared figures, and drafted the manuscript. G.F.S., G.G., A.P., G.D., C.B., and M.V. performed experiments, revised the manuscript, and approved the final version of the manuscript.

## FUNDING INFORMATION

This manuscript has been partially supported by the International Visiting Professor Grant of the University of Verona #1643.

## CONFLICT OF INTEREST STATEMENT

The authors declare no conflict of interest.

## ETHICS STATEMENT

The ethical committee approved all procedures of the University of Verona (CARP; acceptance number IRB #27111) and were performed following the Declaration of Helsinki standards. The participants gave written, informed consent before their participation after a full explanation of the purpose and experimental procedures of the study.

## Data Availability

Data will be made available upon request.
